# A 45 min bout of hot‐water immersion induces transient plasma volume contraction without a detectable increase in circulating erythropoietin

**DOI:** 10.1113/EP094285

**Published:** 2026-10-26

**Authors:** Owain Zerilli, Joseph A. Killick, Andrew J. M. Douglas, Dan Nash, Michael G. Hughes, Mike Stembridge, Elliott J. Jenkins

**Affiliations:** ^1^ Cardiff School of Sport and Health Sciences Cardiff Metropolitan University Cardiff UK

**Keywords:** Erythropoietin, heat stress, hot‐water immersion

## Abstract

Heat acclimation is increasingly used to induce haematological adaptations and enhance endurance performance. Although repeated hot‐water immersion (HWI) has been shown to increase haemoglobin mass, the acute physiological responses that might underpin these longer‐term adaptations remain poorly understood. This study investigated the 24 h time course of plasma volume (PV) and erythropoietin (EPO) responses following a single bout of HWI. Sixteen healthy adults (four females) completed a randomised crossover study comprising 45 min of HWI (40°C) and a time‐matched control condition. Changes in PV were estimated from haemoglobin concentration and haematocrit, while serum EPO concentrations were determined using an ELISA. HWI induced a substantial contraction in PV immediately after immersion (−9.5%, 95% CI [−12.5, −6.5]; *P *< 0.001), accompanied by marked increases in tympanic temperature and thermal strain. However, PV returned to baseline within 12 h, and no subsequent PV expansion was observed. Serum EPO concentration was unaffected by time (*P *= 0.183), condition (*P *= 0.125) or the condition × time interaction (*P *= 0.309). Furthermore, changes in PV were unrelated to changes in circulating EPO in both the HWI (β = 0.07; [−1.3, 1.4]; *P *= 0.915) and control (β = 1.10; [−1.1, 3.3]; *P *= 0.326) conditions. These findings demonstrate that a 45 min bout of HWI induces a substantial but transient contraction in PV that is not accompanied by a measurable increase in circulating EPO within 24 h. Acute PV contractions following HWI therefore appear insufficient to stimulate a measurable erythropoietic response, suggesting that the haematological adaptations observed following chronic heat acclimation may instead depend upon sustained alterations in PV arising from repeated heat exposures.

## Abstract (highlights)

- **What is the central question of this study?**
  Hot‐water immersion (HWI) can increase haemoglobin mass following repeated exposure, but physiological mechanisms underpinning this adaptation remain unclear. Can a 45 min bout of HWI elicit an erythropoietin response within 24 h, and are any changes in erythropoietin associated with concurrent alterations in plasma volume (PV)?
- **What is the main finding and its importance?**
  HWI induced PV contraction, but no subsequent PV expansion or measurable increase in circulating erythropoietin within the ensuing 24 h, suggesting that acute volumetric perturbations induced by HWI might be insufficient to stimulate erythropoiesis. Haematological adaptations observed following chronic heat acclimation may therefore depend upon sustained alterations in PV arising from repeated heat exposures.

## 1 INTRODUCTION

Long‐term heat exposure has become an increasingly popular strategy to enhance endurance performance, reflected both in applied practice and in a growing body of literature. Early work has focused primarily on active heat acclimation (exercise performed in environmental chambers or while wearing thermally insulative clothing) as a means of increasing haemoglobin mass (Hb_mass_) when undertaken over 5 weeks (Cubel et al., 2024; Lundby et al., 2023; Oberholzer et al., 2019; Rønnestad et al., 2021; Rønnestad, Lid et al., 2022; Rønnestad, Urianstad et al., 2022). More recently, passive heat exposure, such as hot‐water immersion (HWI), has been shown to induce comparable Hb_mass_ adaptations over a similar time frame (Jenkins, Killick et al., 2025), expanding the practical scope of heat‐based interventions. Despite the consistent efficacy of these interventions, the physiological mechanisms underpinning heat‐induced increases in Hb_mass_ remain unclear. Although several theories have been proposed to explain these haematological adaptations (as reviewed by Jenkins, Edgett et al., 2025), the regulatory processes and temporal dynamics governing this response have not been characterised directly. Consequently, the mechanistic basis of Hb_mass_ expansion following prolonged heat exposure remains incompletely understood.

A leading explanation for this adaptation is the ‘critmeter’ hypothesis, which posits that an initial (heat‐induced) expansion in plasma volume (PV) reduces haematocrit (Hct), thereby stimulating erythropoiesis to restore Hct towards baseline levels (Donnelly, 2001; Dunn & Donnelly, 2007). This temporal sequence has been proposed to underpin the rise in red cell volume observed following longer‐term heat interventions (Jenkins, Killick et al., 2025; Rønnestad, Urianstad et al., 2022). Direct evidence describing the temporal dynamics of erythropoietin (EPO) across these interventions remains limited. For example, Oberholzer et al. (2019) reported an increase in Hb_mass_ following 5 weeks of active heat exposure, despite observing no change in circulating EPO at either the mid‐point or end of the intervention. Such findings suggest that EPO responses to heat exposure might be transient and occur outside commonly sampled time points, raising the possibility that a single heat exposure might evoke an acute erythropoietic signal. Indeed, DiMarco et al. (2024) reported a significant elevation in EPO within 6 h of a single 45 min bout of HWI, although this response was driven by female participants and was attributed to transient renal hypoperfusion during heating. Notably, PV changes were not assessed, leaving unresolved whether concurrent haemoconcentration or haemodilution might have contributed to the observed EPO response. Experimental manipulation of PV via plasmapheresis has been shown to increase EPO concentration within 24 h (Roberts et al., 2000), suggesting that acute PV contraction might influence erythropoietic signalling, although the transient removal of red blood cells prior to reinfusion makes it difficult to isolate the independent effect of PV changes. Acute PV contraction might also promote sodium‐retaining responses that increase renal oxygen consumption through enhanced tubular sodium reabsorption (Kiil et al., 1961), thereby reducing local oxygen availability and potentially stimulating EPO synthesis (Jenkins, Edgett et al., 2025). These findings highlight uncertainty not only regarding the mechanisms linking heat stress, PV and erythropoiesis, but also the precise time course over which EPO responds to passive heating and associated volumetric perturbations.

Accordingly, the present study aimed to investigate the 24 h time course of EPO and PV responses following a single bout of HWI. We hypothesised that HWI would elicit an acute perturbation in PV, characterised by transient contraction and subsequent expansion, alongside an increase in circulating EPO during the 24 h recovery period.

## 2 MATERIALS AND METHODS

### 2.1 Ethical approval

Study procedures were approved by the Cardiff School of Sport and Health Sciences Ethics Committee (PGT‐9063) and conformed to the ethical standards established by the *Declaration of Helsinki* (2013), excluding prior registration in a database. All participants completed a physical activity readiness questionnaire and provided written informed consent prior to participation.

### 2.2 Participants

An a priori sample size estimation was performed using G*Power (v.3.1.9.4; Heinrich Heine University Düsseldorf, Düsseldorf, Germany). Given the limited literature examining acute EPO responses to HWI, the expected effect was informed by DiMarco et al. (2024), who reported an increase in circulating EPO following a comparable single‐bout HWI protocol. Based on a repeated‐measures design, α = 0.05, power = 0.80 and an assumed effect size (*f* = 0.4), a minimum sample size of 16 participants was required. Seventeen participants were recruited initially; however, one participant was unable to complete both experimental conditions. Therefore, data are presented for 16 participants (12 male and 4 female; age, 28 ± 5.8 years; mass, 64.6 ± 11.4 kg; and height, 172.25 ± 10.26 cm). All participants identified as moderately to highly active, reporting at least four exercise sessions per week and a weekly training volume of 6.7 ± 2.5 h.

Given that EPO synthesis and heat tolerance have been shown to differ across stages of the menstrual cycle (Tenaglia et al., 1999; Yasuda et al., 2001), visits undertaken by eumenorrhoeic female participants (*n* = 2) were scheduled during the early follicular phase, as defined by Elliott‐Sale et al. (2021), and repeated during the same menstrual phase in a subsequent cycle. The participant using continuous oral hormonal contraception (*n* = 1) completed visits 1 week apart, whereas the participant using cyclical oral hormonal contraception (*n* = 1; 3 weeks of use, 1 week of withdrawal) completed both visits during their withdrawal phase.

### 2.3 Study design

The study used a randomised crossover design, with participants completing both HWI and time‐matched control conditions. Each condition lasted 45 min, with visits separated by a minimum of 7 days to allow sufficient washout between trials (Périard et al., 2016). Participants attended the laboratory at the same time of day for both conditions (06:00–08:00 h) and were instructed to arrive well hydrated. Both conditions were completed in an ambient temperature of ∼20°C, with HWI performed in a dedicated bath room and CON completed as posture‐matched rest in a temperature‐controlled laboratory.

### 2.4 HWI

HWI was performed using a protocol consistent with previous work (Jenkins, Killick et al., 2025; Jenkins et al., 2026). Briefly, immersion was completed in a bath initially heated to 40°C and maintained at >39°C throughout. Participants were submerged to shoulder level for the first 15 min. Thereafter, participants were permitted to adjust their position within the bath and expose their arms and torso as required to manage heat tolerance, but remained in the bath for the full 45 min session. Participants were given 500 mL water to drink during the immersion. Tympanic temperature was assessed every 15 min as an indirect index of thermal strain, alongside thermal comfort and sensation using scales adapted from Gagge et al. (1967). Body mass was measured pre‐ and post‐immersion to quantify absolute fluid loss (in grams), dehydration (fluid lost relative to initial body mass) and sweat rate (in litres per hour).

### 2.5 Blood sampling

An 8.5 mL venous blood sample was obtained prior to each condition. Subsequent samples were collected immediately post‐condition and at 6, 12 and 24 h following cessation. Samples were allowed to clot at room temperature for a minimum of 30 min before centrifugation at 1300*g* for 10 min, in accordance with manufacturer guidelines (BD Vacutainer Serum Tube, Wokingham, UK). One thousand microlitres of serum was aliquoted and stored at −80°C until analysis. Serum EPO concentration was determined in duplicate using a commercially available (Invitrogen EPO ELISA, Paisley, UK) with a sensitivity of 0.14 mIU mL^−1^. EPO concentrations are presented as measured values and were not adjusted for concurrent changes in PV.

In addition to venous sampling, capillary blood samples were collected for the determination of Hct and haemoglobin concentration ([Hb]). Hct was determined in triplicate after samples were centrifuged and measured with a micro‐Hct reader (Hawksley, Lancing, UK). [Hb] was analysed in triplicate via blood gas analysis (ABL80 CO‐OX; Radiometer, Copenhagen, Denmark). Percentage changes in PV from baseline were then calculated using the following formula (Dill & Costill, 1974):

$$\Delta \%\mathrm{PV}=\left\{\left(\frac{\left[Hb_{\mathrm{before}}\right]}{\left[Hb_{\mathrm{after}}\right]}\right)\times \left(\frac{1-\left(\frac{\mathrm{Hc}t_{\mathrm{after}}}{100}\right)}{1-\left(\frac{\mathrm{Hc}t_{\mathrm{before}}}{100}\right)}\right)-1\right\}\times 100$$

### 2.6 Statistical analysis

All statistical analyses were performed using GraphPad Prism (v.10.0.3; GraphPad Software, Boston, MA, USA). Statistical significance was accepted at *P* < 0.05. Data are presented as the mean ± SD unless otherwise stated, and between‐condition differences are reported as mean differences with 95% confidence intervals (CI).

Physiological and perceptual responses associated with the HWI intervention were assessed by comparing pre‐ and post‐immersion measurements of tympanic temperature, thermal sensation, thermal discomfort and body mass using paired Student's *t*‐tests. Changes in EPO concentration and PV were analysed using a two‐way repeated‐measures ANOVA, with condition and time specified as within‐subject factors. Where data were missing for a given condition or time point, a mixed‐effects model was used to account for within‐subject variability while retaining all available observations. Significant main effects or interactions were explored using Tukey's *post hoc* tests.

Associations between changes in EPO and PV were assessed using a linear mixed‐effects model in R (v.4.6.1; R Foundation for Statistical Computing, Vienna, Austria). Change in EPO was specified as the dependent variable, with the change in PV, condition and time point and the change in PV × condition interaction included as fixed effects. Participant was included as a random intercept to account for repeated observations within individuals. Condition‐specific slopes were extracted to determine whether the association between changes in PV and EPO differed between HWI and CON.

## 3 RESULTS

### 3.1 Responses to HWI

Physiological and psychophysical responses to the HWI bout are presented in Table 1. Tympanic temperature increased following HWI, rising from 36.1°C to 38.6°C upon exit of the bath (Δ = 2.5°C, 95% CI [2.1, 2.9], *P* < 0.001). Participants experienced a substantial fluid loss of 970 ± 400 mL, corresponding to a sweat rate of 1.3 ± 0.5 L h^−1^. Perceptual markers of heat stress also increased during the HWI, with mean thermal sensation rising from 6.2 to 10.7 (1–13 scale, *P* < 0.001) and mean thermal discomfort rising from 1.3 to 6.7 (1–10 scale, *P* < 0.001) at the cessation of the bath.

**TABLE 1:** Physiological responses to hot‐water immersion.

Parameter	Pre	15 min	30 min	Post	*P*‐value (pre vs. post)
Tympanic temperature, °C	36.1 ± 0.6	37.9 ± 0.8	38.3 ± 0.5	38.6 ± 0.5	<0.001
Thermal sensation (1–13)	6.2 ± 1.4	9.3 ± 0.7	10.4 ± 1.4	10.7 ± 0.8	<0.001
Thermal discomfort (1–10)	1.3 ± 0.6	4.2 ± 0.9	6.1 ± 1.2	6.7 ± 1.3	<0.001
Body weight, kg	65.0 ± 11.4			64.1 ± 11.3	0.008
Sweat loss, mL				970 ± 400	
Sweat rate, L h^−1^				1.3 ± 0.5	

*Note*: Values are presented as the mean ± SD. The *P*‐values represent the effect of a single bout of hot‐water immersion from paired Student's *t*‐tests. Thermal sensation (1 = unbearably cold, 13 = unbearably hot) and thermal discomfort (1 = comfortable, 10 = extremely uncomfortable) were rated using scales adapted from Gagge et al. (1967).

### 3.2 PV responses

PV contracted by 9.5% immediately following HWI (95% CI [−12.5, −6.5]; *P* < 0.001; Figure 1). This reduction was no longer evident at 12 h post‐HWI, with PV returning to baseline values (1.1%, 95% CI [−4.5, 4.1]; *P* > 0.999). No subsequent PV expansion was observed, with values remaining unchanged at 24 h post‐HWI (−1.6%, 95% CI [−5.2, 3.0]; *P* = 0.926). PV did not change across any time point during the control conditions.

**FIGURE 1:**
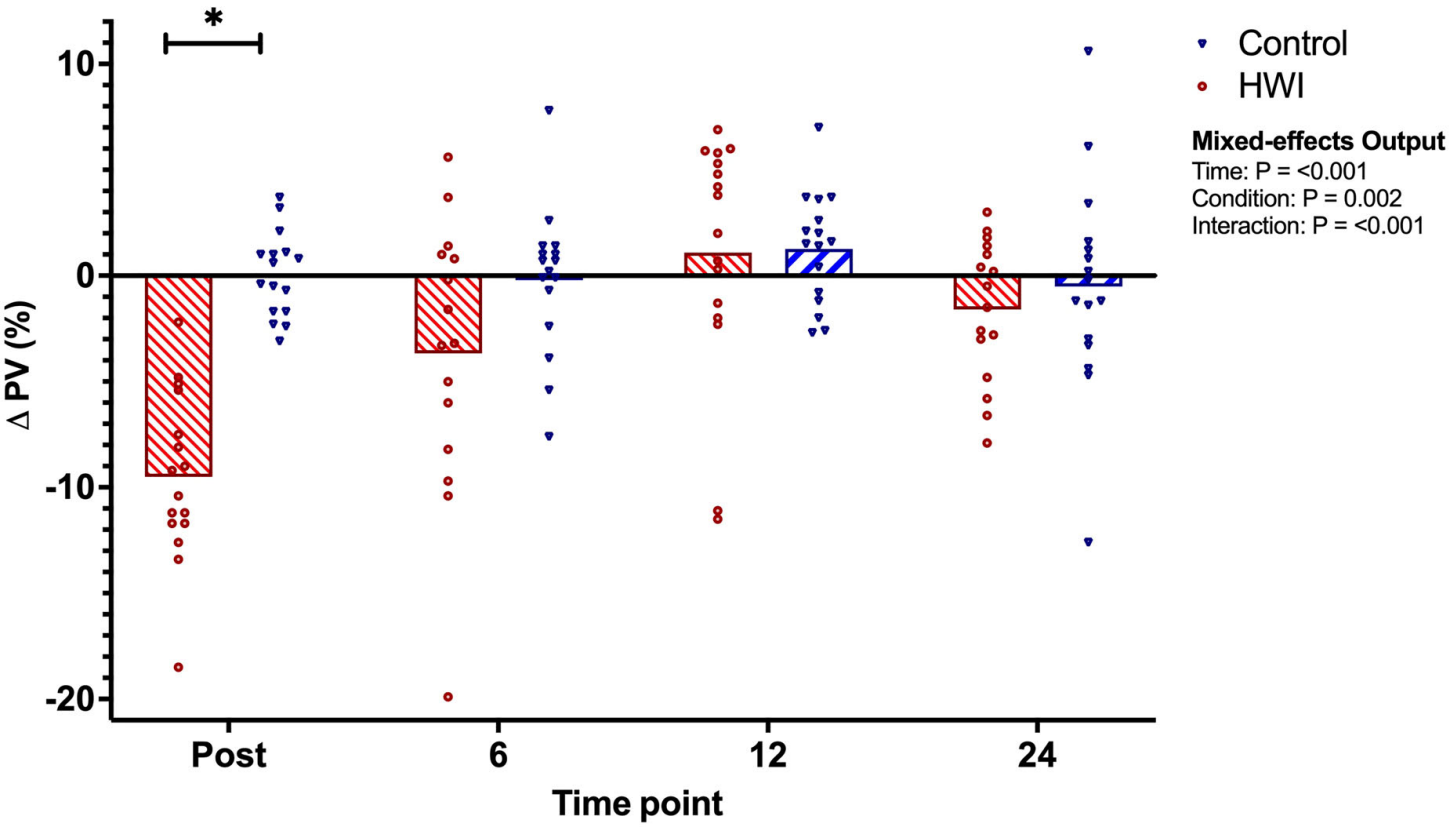
Relative changes in plasma volume (PV) from baseline following hot‐water immersion (HWI) and time‐matched control (CON). Bars represent mean responses, and symbols represent individual participant data. *Significant condition difference at the corresponding time point (*P* < 0.05).

### 3.3 EPO responses

Serum EPO concentrations were unaffected by time (*P* = 0.183), condition (*P* = 0.125) or the condition × time interaction (*P* = 0.309; Figure 2). Furthermore, changes in PV were not associated with changes in circulating EPO in either the CON (β = 1.10; 95% CI [−1.1, 3.3]; *P* = 0.326) or HWI (β = 0.07; 95% CI [−1.3, 1.4]; *P* = 0.915) conditions (Figure 3).

**FIGURE 2:**
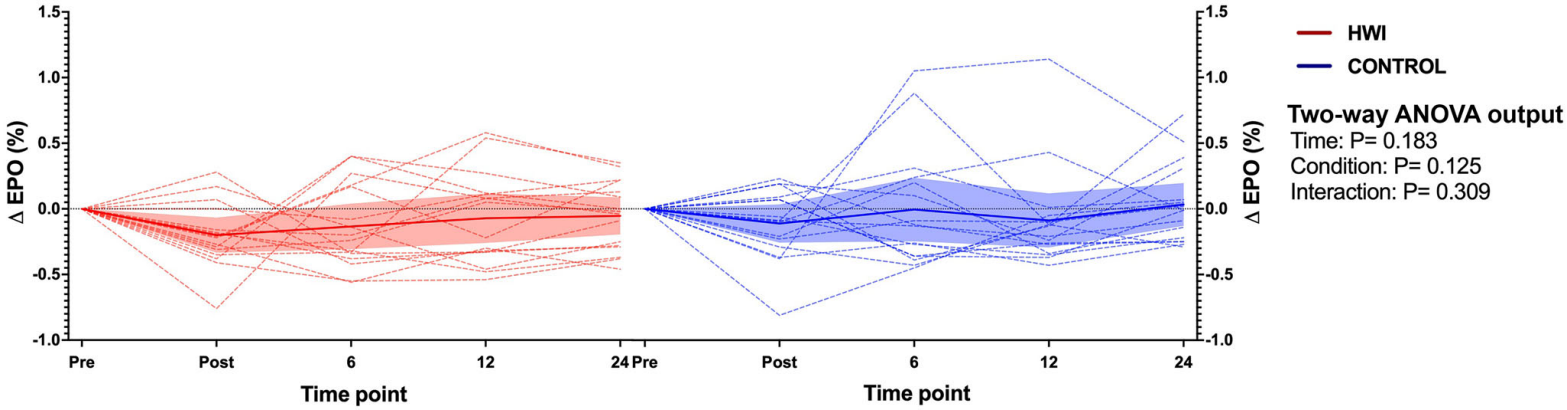
Relative changes from baseline in circulating erythropoietin concentration (∆EPO) following hot‐water immersion (HWI) and time‐matched control (CON). Thin lines represent individual participant responses across time, and the shaded region represents the group mean and 95% confidence interval.

**FIGURE 3:**
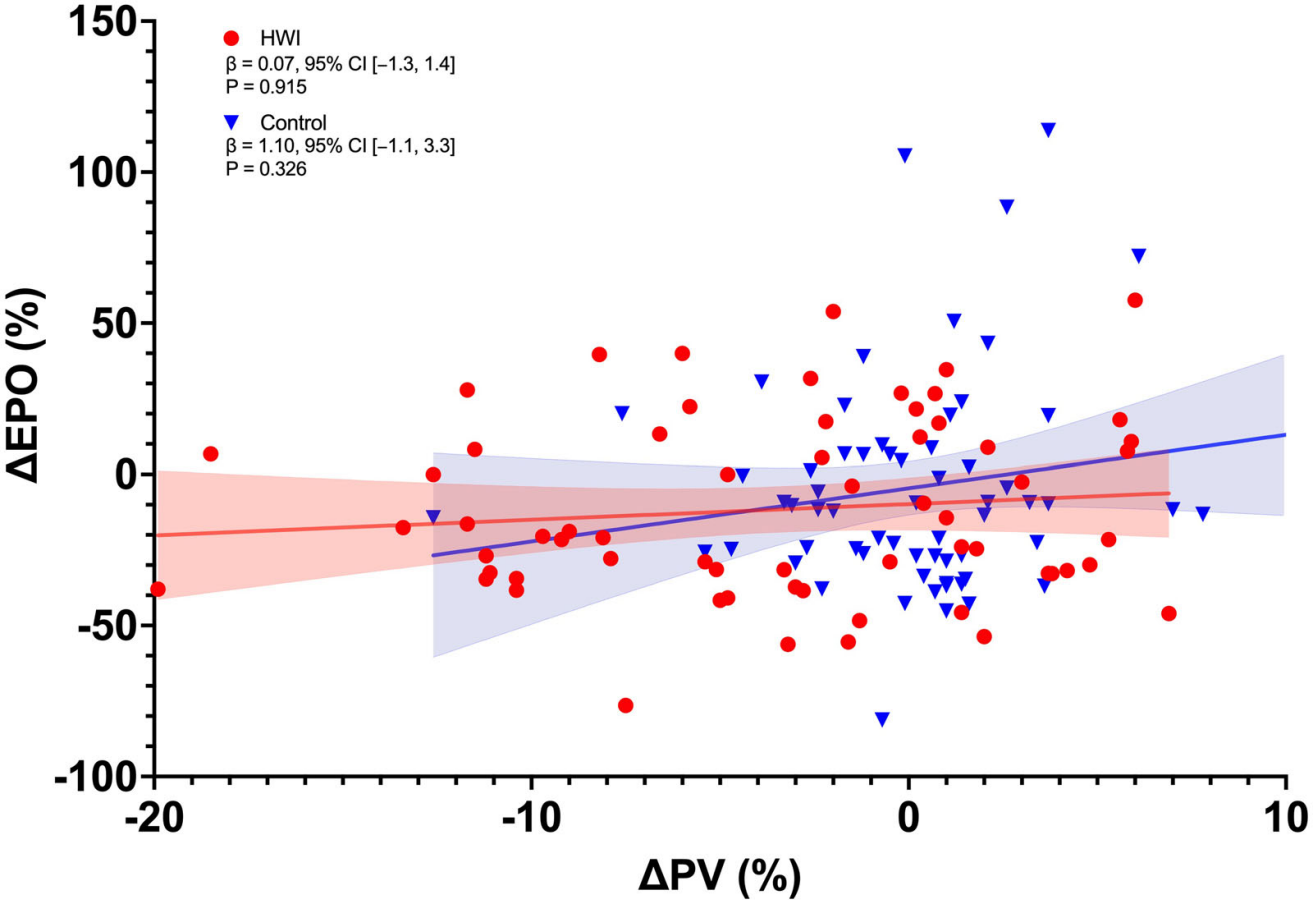
Relationship between changes in plasma volume (∆PV) and circulating erythropoietin concentration (∆EPO) following hot‐water immersion (HWI) and time‐matched control (CON). Continuous lines represent modelled condition‐specific associations from a linear mixed‐effects model, with participant included as a random intercept to account for repeated observations within individuals and time point included as a fixed effect. Shaded regions indicate 95% confidence intervals.

## 4 DISCUSSION

In the present study we sought to investigate the 24 h time course of PV and EPO responses following a 45 min bout of HWI at 40°C. The principal findings were that HWI induced a substantial contraction in PV immediately following immersion, but did not elicit a subsequent PV expansion or measurable increase in circulating EPO within the ensuing 24 h. Furthermore, there was no evidence of an association between changes in PV and EPO concentration, suggesting that the acute volumetric perturbations induced by a 45 min bout of HWI appear insufficient to stimulate a measurable erythropoietic response.

### 4.1 Acute EPO responses to HWI

The present findings differ from those reported by DiMarco et al. (2024), who observed a group‐level increase in EPO within 6 h of HWI and attributed this response to transient reductions in renal blood flow during heating. However, their study did not include control conditions, PV changes were not assessed, and reductions in renal blood velocity were not associated with the observed EPO response, making it difficult to determine the specific stimulus underpinning this response. The HWI protocol used by DiMarco et al. (2024) was broadly comparable to that used in the present study, with both involving 45 min of HWI at ∼40°C. However, the protocols were not identical, and differences in immersion depth, thermometry, participant characteristics and sex distribution might have contributed to the divergent EPO responses. In particular, when analysed separately, male participants in the study by DiMarco et al. (2024) did not exhibit a signifiant increase in EPO, a finding consistent with the present predominantly male cohort. Taken together, these comparisons suggest that acute HWI might increase EPO in some conditions, but that this response is not observed consistently across cohorts or protocols and was not detected following the protocol used in the present study.

The present results provide little support for the notion that acute PV contraction independently stimulates EPO production. Although Roberts et al. (2000) reported an increase in EPO following plasmapheresis‐induced hypovolaemia, the transient removal of red blood cells inherent to that model complicates attribution of the response solely to changes in PV. In the present study, PV contracted by 9.5% immediately following HWI, yet no increase in EPO concentration was observed, and changes in PV were not associated with changes in EPO (Figure 3).

The absence of a measurable EPO response also challenges proposed renal oxygen‐dependent mechanisms linking acute heat stress to erythropoietic signalling. Acute PV contraction has been proposed to increase renal oxygen consumption via sodium‐retaining responses and enhanced tubular sodium reabsorption, thereby reducing local oxygen availability and potentially stimulating EPO synthesis (Jenkins, Edgett et al., 2025). However, the absence of a detectable EPO response despite substantial PV contraction suggests that any reduction in renal oxygen availability induced by a single bout of HWI was insufficient to elicit measurable erythropoietic signalling. Renal blood flow might also have decreased during heating, reflecting the redistribution of blood flow towards the skin for thermoregulation (Chapman et al., 2021; DiMarco et al., 2024); however, any associated reduction in renal perfusion was likewise insufficient to stimulate an EPO response. Although direct measures of renal oxygenation, sodium retention and renal blood flow were not obtained, these findings suggest that acute perturbations in PV and associated renal responses appear insufficient, in isolation, to stimulate EPO production following a single bout of HWI.

### 4.2 Implications for the critmeter hypothesis

Although the present study was not a direct test of the critmeter hypothesis, the absence of an EPO response following HWI warrants consideration in relationship to this proposed mechanism (Donnelly, 2001; Dunn & Donnelly, 2007). According to this hypothesis, sustained plasma volume expansion reduces Hct, thereby creating a stimulus for subsequent erythropoiesis. In contrast, in the present study we observed only an acute PV contraction, with values returning to baseline within 12 h and no evidence of subsequent expansion. The absence of PV expansion is notable given previous reports of hypervolaemia following immersion in both hot and thermoneutral water (Campbell et al., 2022). Differences in post‐immersion hydration, free‐living activity patterns and other behavioural factors might explain these discrepancies in part, although the precise reasons remain unclear. Regardless, the sustained volumetric changes proposed to initiate the critmeter response were not observed in the present study. Nevertheless, the critmeter hypothesis remains a leading explanation for the chronic haematological adaptations observed following repeated HWI. Jenkins, Killick et al. (2025) observed an early expansion in PV, accompanied by reduced Hct, before Hb_mass_ increased later in the 5 week intervention. Likewise, Oberholzer et al. (2019) reported an inverse relationship between Hct and EPO during a 5 week active heat intervention, suggesting that reductions in Hct might be associated with greater erythropoietic signalling. Together, these observations are consistent with the sequence of events proposed by the critmeter hypothesis. However, direct evidence linking PV expansion, reductions in Hct and subsequent increases in EPO remains lacking. Taken together, the present findings indicate that acute fluctuations in PV and Hct following a single bout of HWI do not appear sufficient, in the conditions tested, to elicit a measurable EPO response, while remaining consistent with the possibility that longer‐term volumetric adaptations remain central to the erythropoietic response observed during chronic heat acclimation.

### 4.3 Limitations

Although the present study provides insight into the acute erythropoietic and volumetric responses to HWI, several limitations should be acknowledged. First, thermoneutral‐water immersion conditions were not included. Previous work has demonstrated that immersion alone can alter PV (Campbell et al., 2022) and might influence endocrine responses (Chudek et al., 1997). Consequently, the present design does not allow the independent effects of hydrostatic pressure and heat stress to be disentangled fully. Inclusion of thermoneutral‐water conditions in future studies would help to clarify the specific contribution of heating to acute haematological responses. Second, core temperature was not measured directly. Although indirect, tympanic temperature, thermal sensation and thermal discomfort indicated that the HWI protocol elicited a substantial heat strain, and the protocol itself has been used in previous work demonstrating Hb_mass_ expansion following repeated HWI (Jenkins, Killick et al., 2025). Nonetheless, future work could determine whether the magnitude and duration of core temperature elevations during HWI, which might be influenced by exposure duration, water temperature, immersion depth or preceding exercise, are associated with subsequent EPO responses. Third, it is possible that a transient increase in EPO occurred between sampling windows. However, given that the reported circulating half‐life of EPO is ∼7–8 h (Bunn, 2013), and no increase was observed at any measured time point, a large undetected response appears unlikely. Nevertheless, future studies using more frequent sampling during early recovery could better define the time course of any transient EPO response. Finally, although a substantial contraction in PV was observed immediately following HWI, no subsequent PV expansion was detected. This finding contrasts with previous reports of post‐immersion hypervolaemia (Campbell et al., 2022; Kissling et al, 2019) and might have influenced the interpretation of the critmeter hypothesis. Participants were permitted to resume normal daily activities between measurement time points, and hydration status was not prescribed or monitored. Differences in fluid intake, activity patterns and other behavioural factors might therefore have contributed to the absence of a measurable PV expansion. Future studies would benefit from greater control of hydration and recovery behaviours during the post‐immersion period, alongside objective confirmation of pre‐intervention hydration status using urine specific gravity.

### 4.4 Future directions

Despite growing evidence that repeated heat exposure increases Hb_mass_, the timing of the accompanying EPO response remains poorly understood. The present findings suggest that a 45 min bout of HWI is insufficient to elicit a measurable increase in circulating EPO within 24 h; however, whether EPO increases outside this window or only after repeated heat exposures remains unknown. Future studies should therefore incorporate more frequent sampling across the early stages of repeated heat exposure to define the time course of the EPO response better. Such work would help to determine whether the proposed sequence of PV expansion, reduced Hct and subsequent EPO production occurs during heat acclimation, providing a more direct test of the critmeter hypothesis.

Future work should also seek to identify the physiological factors that regulate PV and EPO responses during heat acclimation. Hydration status (Akerman et al., 2017), renal haemodynamics (DiMarco et al., 2024), recovery posture and central venous pressure (Montero et al., 2016) have all been proposed as potential modulators of blood volume regulation and erythropoietic signalling, yet how these factors influence the PV and EPO responses to heat acclimation remains unclear. Experimental manipulation of these factors, alongside direct measurement of the associated physiological responses, might help to establish the mechanisms responsible for the sustained haematological adaptations observed following chronic heat acclimation. Recent work by Mazza et al. (2026), manipulating central venous pressure through head‐up tilt, provides an example of how targeted changes in haemodynamic loading might help to establish causal links between blood volume regulation and erythropoiesis; similar approaches applied within active and passive heat protocols might help to determine whether altering the post‐heating haemodynamic environment can modify PV expansion, EPO signalling and subsequent haematological adaptation.

## 5 CONCLUSION

The present study characterised the 24 h time course of EPO and PV responses following a 45 min bout of HWI at 40°C. Although HWI induced a substantial contraction in PV, no subsequent PV expansion or detectable increase in circulating EPO was observed within the ensuing 24 h. Furthermore, there was no evidence of an association between changes in PV and EPO concentration, suggesting that acute volumetric perturbations induced by a 45 min bout of HWI appear insufficient to stimulate a measurable erythropoietic response. These findings provide new insight into the acute haematological response to HWI and suggest that the erythropoietic adaptations observed following chronic heat acclimation may instead depend upon sustained alterations in PV arising from repeated heat exposures.

## AUTHOR CONTRIBUTIONS

All authors contributed to the design and writing of the manuscript. All authors approved the final version of the manuscript and agree to be accountable for all aspects of the work in ensuring that questions related to the accuracy or integrity of any part of the work are appropriately investigated and resolved. All persons designated as authors qualify for authorship, and all those who qualify for authorship are listed.

## CONFLICT OF INTEREST

None declared.

## FUNDING INFORMATION

No funding was received for this work.

## GENERATIVE AI STATEMENT

During the preparation of this manuscript the authors used ChatGPT (OpenAI) to support language editing, clarity, structure and refinement of author‐generated text. The tool was not used to generate original scientific ideas, analyse data, create figures or produce references. All AI‐assisted suggestions were reviewed, edited and approved by the authors, who take full responsibility for the content of the submitted manuscript.

## Data Availability

The datasets generated and analysed during the study are available from the corresponding author upon reasonable request.
